# Adjuvant Aromatase Inhibitors in Early Breast Cancer May Not Increase the Risk of Falls

**DOI:** 10.4172/2469-6684.100023

**Published:** 2016-06-07

**Authors:** Palak Choksi, Margaret E Williams, Kelley M Kidwel, Julia Stella, Mary Soyster, David Hanauer, Catherine Van Poznak

**Affiliations:** 1 Department of Internal Medicine, University of Michigan Health Systems, USA; 2 Department of Biostatistics, School of Public Health, University of Michigan, USA; 3 University of Michigan Medical School, USA; 4 University of Connecticut School of Medicine, USA; 5 Department of Computational Medicine and Bioinformatics, University of Michigan Medical School, USA

**Keywords:** Postmenopausal women, Aromatase inhibitors, Early stage breast cancer, Falls, Fractures

## Abstract

**Background:**

Falling increases the risk for fracture. The impact of adjuvant aromatase inhibitors (AI) on the risk of falls is undefined.

**Methods:**

A retrospective case control study was performed examining women with early stage breast cancer on adjuvant AI and matched controls without cancer. Fall and fracture data were abstracted from the medical record.

**Results:**

Matched pairs of 332 women were identified (total N = 664). There was no statistically significant difference in the odds of a fall between cases and controls, p = 0.86. Similarly, the odds of a fracture between cases and controls was not significantly different, p = 1.0. There were 35 pairs in which the case fractured but the control did not and equal number of pairs where the control fractured but the case did not. For pairs in which control fractured but case did not, the median age at fracture was significant higher than that for pairs in which case fractured but the control did not (71 *vs.* 63 years p = 0.0003).

**Conclusion:**

This study did not identify a difference in the incidence of falls or fractures in women on adjuvant AI compared to their age matched controls without breast cancer. Prospective studies of falls and fracture in women on adjuvant AI therapy compared to age match controls would aid in the identification of fracture risk.

## Introduction

Breast cancer and osteoporosis are both common diagnoses in women. Approximately 12% of women in the US will develop invasive breast cancer [1] and approximately 10% of women over the age of 50 will experience an osteoporotic fracture in their lifetime [2]. Adjuvant therapy used in the management of breast cancer, such as the aromatase inhibitors, promotes bone loss and increases the risk for fracture [3]. Fractures are associated with significant morbidity, mortality and health care expenditure [4]. As breast cancer affects over 200,000 women annually in the US [5] the treatment associated toxicities such as osteoporosis pose a true public health concern. The ten-year cancer free survival for stage I–III breast cancer is 80% [6]. Understanding fall and fracture risk plays an important role in preserving the health, independence, and quality of life of individuals on active treatments as well as the survivors.

Approximately 75% of postmenopausal breast cancers express either estrogen or progesterone receptors and often called hormone receptor positive (HR+) [7]. When compared with tamoxifen, in postmenopausal women with HR+ tumors, aromatase inhibitors (AIs) are the treatment of choice as they further reduce the incidence of recurrence [8]. The depletion of circulating estrogen produced by AIs is associated with an increase in osteoclast activity and osteoblast apoptosis, resulting in accelerated bone resorption, loss in bone mineral density (BMD) and increased risk for fractures. Breast cancer survivors are at an increased risk of fractures [9]. At three years post treatment initiation, approximately 10% of women who are treated with an AI will develop a fracture [10]. The Women’s Health Initiative study has shown that post-menopausal women diagnosed with breast cancer had a higher risk of hip fracture than did their counterparts without breast cancer [11].

AI associated arthralgia, affects approximately 40% to 50% of patients and often develops within the first 6 months of AI initiation [12, 13]. Pain, such as that associated with arthritis has been associated with an increased risk for falling and fracture [14]. It is unknown whether the use of adjuvant AI therapy is associated with an increased risk for falls.

The primary objective of this study was to generate data on the reported prevalence of falls and fractures in postmenopausal women treated for early stage breast cancer with an AI compared to matched controls who are cancer free and not exposed to an AI. Although falls are a significant risk factor for fractures, there is insufficient data on the proportion of patients on AIs who fall, therefore this retrospective study was designed to explore falls in this population. In this case control study, we hypothesized that women treated for breast cancer with an AI have a higher risk of falling compared to women without breast cancer and without AI exposure. We also examined prevalence of osteopenia and osteoporosis in these two groups and use of calcium and bisphosphonates.

## Methods

The University of Michigan (UM) Health System Electronic Medical Records Search Engine (EMERSE) the UM Health System (UMHS) data warehouse, the UM Cancer Registry and Institutional Review Board (HUM00063088) approved this retrospective study [15]. UMHS provides approximately two million outpatient appointments annually and primarily serves the three nearby counties in south-eastern Michigan.

The cases were defined as postmenopausal women who have received an adjuvant AI (anastrazole, exemestane or letrozole) for HR+ breast cancer. Cases were identified using the EMERSE and the UM Cancer Center Registry. The search identified 332 postmenopausal women with early-stage breast cancer treated with an AI initiating therapy between the years 2004–2007, with five year follow up. Records past 2012 were excluded due to the change in electronic medical record formatting. These women represent the case cohort. Controls were defined as not having cancer and thus not exposed to an aromatase inhibitor in the same time frame. In addition, controls were required to have at least three separate UMHS medical encounters to ensure adequate follow up.

Using the UM Health Systems data warehouse 674,072 controls were identified. Potential controls were race and age matched based where age of the case was defined as the time of breast cancer diagnosis +/−30 days (hence age at fall or fracture may differ). The first alphabetically listed control that met the criteria was selected for study inclusion. One control was selected for each of the 332 cases. The study population consists of 332 matched pairs comprising of cases (postmenopausal women with HR+ early-stage breast cancer taking AIs) and matched controls with a total sample size of 664.

Using EMERSE, data were abstracted from the medical record for both the controls and cases. Data abstracted was limited to the study specific time frame. Age at first fall, age at first fracture, femoral neck T score measured via dual energy X-ray absorptiometry (DXA), diagnosis of osteopenia or osteoporosis, use of calcium and use of bisphosphonate were abstracted from the time of initiating an AI to 3 months after stopping an AI and during the same period for controls. The subjects were followed for a period of 5 years. Data was compiled for each pair of cases and controls.

The association between case and control status and the rate of falls and fractures was assessed with conditional logistic regression models accounting for the matched data. Age at time of first fall or first fracture between pairs where cases or controls fell or fractured but the other cohort did not was compared using a two-sample t-test. For pairs in which both the cases and control fell and fractured, the difference in age at first fall or fracture was calculated and analyzed using a sign test. The association between case or control status and calcium use was assessed using McNemar’s test. Within the AI users (cases), chi-square tests were used to investigate the association between bisphosphonate use, falls and fractures. Analysis was completed using SAS v9.3.

## Results

The median age of the 664 postmenopausal women serving as cases and controls was 67 years (range 34–95). The majority (91.9%) of patients were white with 4.5% African American and 3.6% other or unknown race (Table 1).

### Falls

**Figure 1:**
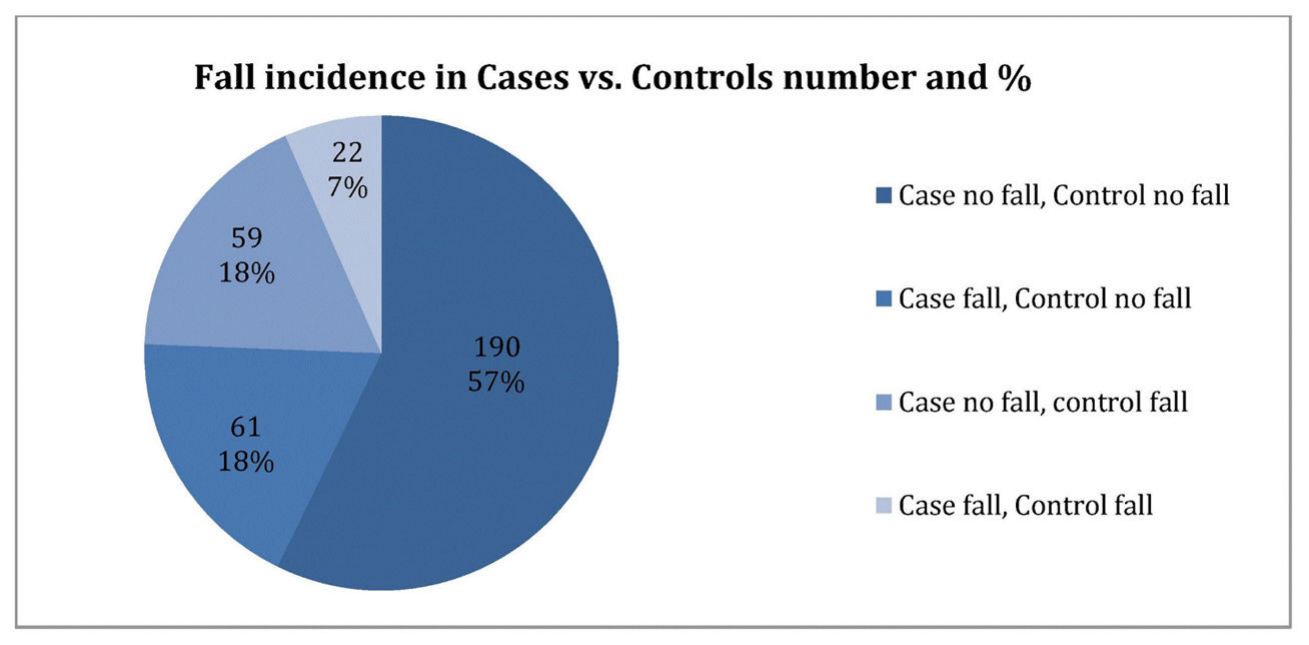
Frequency of case and control pairs with falls. There is no statistically significant difference in the odds of falls between cases and controls, p = 0.86.

In the entire study population 164 (24.7%) patients experienced a fall within the five-year study period (83 cases 25.0% *vs.* and 81 controls 24.4%). For the matched pairs, there were 190 pairs (57.2%) where neither the case nor control fell. In 61 pairs (18.4%) the case fell but the control did not. In 59 pairs (17.8%) the control fell but the case did not. There was no statistically significant difference in the proportion of women who experienced at least one fall in age-matched cases and controls, p = 0.86 (OR 1.03, 95% CI 0.72–1.48). Figure 1 illustrates the proportion of women who experienced at least one fall.

### Age at first fall

The age at first fall was missing for 3 controls. For the 61 pairs in which the case fell but control did not, median age at first fall during the time period studied was 64 years (range of 37–90 years), compared with median age of 67 years (range of 35–95 years) for pairs in which control fell but case did not. Although numerically the women on AI therapy experienced their first on study fall at a younger age, this was not statistically significant, p = 0.17. For the 22 pairs in which both case and control fell there was no difference in the age at time of fall, p = 0.19. Specifically, in those pairs where both case and control experienced falls, the median age at time of fall was 71 years (range 49–91 years) for cases and 71 years (range 45–91 years) for controls.

### Fractures

**Figure 2:**
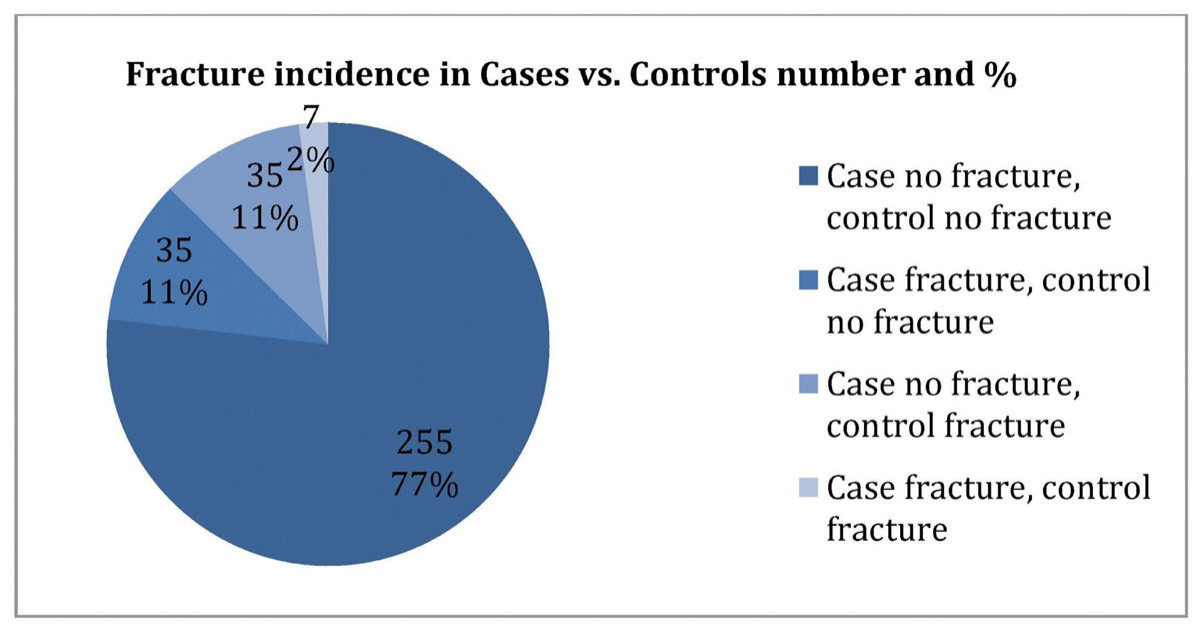
Frequency of case and control pairs with fractures. There is no statistically significant difference in the odds of fractures between cases and controls, p = 1.0.

Approximately 84 women (12.7%) of the entire study population experienced a fracture within the 5-year study period (42 cases (12.7%) and 42 controls (12.7%). Accounting for matching, there were 255 pairs (76.8%) where neither the case nor control had a fracture. In 35 pairs (10.5%) the case experienced a fracture but the control did not, and there were an equal number of pairs (35) where the control experienced a fracture but the case did not (Pairs data shown in Figure 2). There were 7 pairs (2.1%) where both the case and control experienced a fracture. There was no statistically significant difference in the proportion of women with fractures in age-matched cases and controls, p = 1.0 (OR 1.00, 95% CI 0.63–1.60).

### Age at first fracture

The age at the time of fracture was missing for 7 controls and for 1 case. For pairs in which the case fractured but control did not, the median age of fracture was 63 years (range 37–80) which was significantly younger than pairs in which control fractured but case did not (median age at fracture was 71 (range 51–91 years, p = 0.0003). From the available data on the age of fracture, in pairs where both the case and control experienced a fracture, the median age of the case at fracture (n = 7) was 73 years and for controls (n = 6) was 67. However this difference is not significant (p = 0.22) is limited by low power.

### BMD

Data on the presence or absence of osteopenia/osteoporosis as ascertained via DXA was not available for 327 (49.2%) of the 664 women in this study. BMD was available in 259 cases (78.0%) and only 78 controls (23.5%). Due to the low number of BMD results available, especially for controls, no statistical comparisons could be made on T scores or the presence of osteopenia or osteoporosis between cases and controls or incidence of fracture with relation to BMD.

### Calcium and bisphosphonate use

Medical records were reviewed to determine use of calcium and bisphosphonates. Data on calcium supplementation in 5 pairs was not available, thus 322 pairs were analyzed for associations with calcium. Data on the use of bisphosphonate was not available for one pair, thus 331 pairs were analyzed for associations with bisphosphonates.

In 99 pairs (30.7%) both cases and controls reported use of calcium supplements. In 48 pairs, both case and control reported that they did not use calcium. There were significantly more pairs where the case was taking calcium supplements than the controls (p < 0.0001). Specifically, there were 134 pairs (41.6%) where cases reported use of calcium supplements but the controls did not and in 41 pairs (12.7%) where the controls reported use of calcium supplements but case does not. Calcium supplementation was not significantly associated with the odds of a fall, p = 0.16 (OR 1.46, 95% CI 0.86–2.47). In addition, when controlling for calcium use, neither case nor control status, was significantly associated with the odds of a fall (OR 0.87, 95% CI 0.59–1.27, p = 0.54). Calcium supplementation was borderline statistically significant in the increased odds of fracture, p = 0.05 (OR 2.24, 95% CI 0.99–5.10). Controlling for calcium use, there was no statistically significant difference between cases and controls with the odds of fracture (OR 0.68 95% CI 0.40–1.18, p = 0.17).

There were 16 pairs (4.8%) where both cases and controls were taking bisphosphonates, and 191 pairs (57.7%) where neither case nor controls were taking a bisphosphonate. There were significantly more pairs where the case received bisphosphonates and the control did not, p < 0.0001. Specifically, there were 102 pairs (30.8%) where the case received a bisphosphonate but the control did not and only 22 pairs (6.6%) where the control received a bisphosphonate but the case not. Taking a bisphosphonate was not significantly associated with the odds of a fall (OR 1.43, 95% CI 0.81–2.51, p = 0.22). Controlling for the use of bisphosphonates, there was no difference in the odds of fall between cases and controls (OR 0.97, 95% CI 0.67–1.41, p = 0.88). Similarly taking a bisphosphonate was not significantly associated with odds of fracture, p = 0.11 (OR 2.00, 95% CI 0.86–4.66) and controlling for bisphosphonate use, there was not a significant difference between cases and controls in the odds of a fracture, p = 0.64 (OR 0.89, 95% CI 0.54–1.46). In the subgroup of women who took an AI (n = 332 the cases), use of bisphosphonates was not significantly associated with fractures (p = 0.29), but there was a significant association between taking bisphosphonates and falls, p = 0.025. Specifically, taking a bisphosphonate was significantly associated with an almost two-fold increase in the odds of a fall (OR 1.78, 95% CI 1.07–2.96).

## Discussion

This retrospective case-control study of 332 matched pairs demonstrated that postmenopausal women with breast cancer on adjuvant AIs did not have an increased risk of fall or fracture as compared to similar women without cancer who were not on an AI. However, the median age of those AI patients fractured was significantly younger (difference in median ages of 8 years) than the controls that fractured. There was no difference in age among the women on an AI who fell compared to matched controls.

This novel data suggest that although the frequency of falls and fractures did not differ significantly between cases and controls, the women on AI therapy were receiving a more intensive bone health regimen than the controls as noted by the greater number of women undergoing BMD testing and the use of calcium supplements and bisphosphonates. Approximately three times as many cases had undergone BMD testing than their matched controls. The heightened awareness for AI therapy linked with the risk of bone loss and fracture was likely the factor promoting this difference in BMD testing and treatment [16, 17].

The retrospective design of this study does not permit full assessment of risk for fracture or falls, or evaluating the reason so few controls had BMD data, particularly given that the median age was 67 and BMD testing is recommended for women over the age of 65 [18]. The reporting of falls, fractures, BMD and use of bisphosphonate and calcium depended on the patients receiving care through UMHS. Data generated may have been influenced by patient reports of these factors at their appointments and care providers recording the events in the medical record. Scanned documents from outside hospitals were also reviewed in this analysis, but such data is dependent on the outside hospital records being sent to UMHS. Patient reporting may also have been a factor influencing the data.

Although the retrospective study design is associated with limitations in capturing events, that limitation is equal across the cases and controls. The number of falls, data on fracture, the use of calcium supplements and bisphosphonates may be under reported in this study. Each year approximately one third of adults over the age of 65 fall although only less than half discuss the fall with their health care provider [19, 20]. For this study population the median age is 67 years and 25% of subjects experienced a fall, which is consistent with what may be expected based on Center for Disease Control estimates. This study did not assess for comorbid conditions affecting the risk of falls or fractures, including neuropathy. As UMHS is a tertiary care center, the population of patients tends to have multiple medical issues and thus may be more at risk of falls and fractures than the general population, and perhaps this could narrow the differences in fall and fracture between AI patients and their matched controls in this study.

Our data are novel, as our comparator group did not have cancer or history of previous AI use. Compared to tamoxifen, AI are associated with a greater risk of loss of BMD and fracture [8]. Tamoxifen, a selective estrogen receptor modulator has been shown to maintain or increase BMD by 0.5–1.0% per year in postmenopausal women [21]. Hence it is possible that the negative impact of adjuvant AI therapy is further skewed by the comparison to tamoxifen. This current study did not include postmenopausal women treated with adjuvant tamoxifen and cannot speculate on a potential third cohort.

A prospective observational pilot study of postmenopausal women under the age of 70 with a history of breast cancer on adjuvant chemotherapy and/or endocrine therapy reported that 58% study participants reported a fall within the last year and 53% experienced one or more falls during the six-month follow up period [22]. The average age of study participants was 58 and less than 10% had a fracture after their breast cancer diagnosis, although the fractures that did occur were commonly associated with a fall [22]. A separate report of a multifactorial examination of cancer survivors 55 years and older who were 1 or more years out from a cancer diagnosis (n = 39) demonstrated that 56% of participants reported at least one fall in the prior 12 months [23]. Peripheral neuropathy may increase the risk of falls and in patients with grade 4 or greater peripheral neuropathy falls have been reported in approximately 12% of patients within a Phase III clinical trial [24]. These data are relatively consistent with the findings reported here in this retrospective study where 25% of subjects experienced a fall and 13% experienced a fracture.

The results of this retrospective case control study are consistent with expected falls and fracture data for postmenopausal women receiving adjuvant AI therapy, and generates provocative, novel findings by pairing the AI treated subjects to matched controls. The suggestion that adjuvant AIs may not have a profoundly negative effect on fracture compared to matched controls warrants prospective studies. Greater knowledge to define the risk of falls and fractures, measuring BMD, and appropriate optimization of calcium and other anti-resorptive agents can help protect the health, independence, and mortality of women with breast cancer undergoing active treatment as well as survivors.

## Figures and Tables

**Table 1:** Baseline demographics of the study population.

	Cases	Controls

Age Median (Mean) in years	67 (67.34)	67 (67.30)
Age range in years	34–95	34–95

Duration of AI use (months)	4.14 (3.50)	-
Range	0–7.33	0–7.33

Race	White 305 (91.9%)	White 305
	Black 15 (4.5%)	Black 15
	Other 7 (2.1%)	Other 7
	Unknown 5 (1.5%)	Unknown 5

Bisphosphonate use (number)	Yes: 117	Yes: 37
	No: 214	No: 294

Calcium use (number)	Yes: 236	Yes: 142
	No: 86	No: 180
